# Spatiotemporal changes caused by the intensive use of sea areas in the liaoning coastal economic zone of China

**DOI:** 10.1371/journal.pone.0242977

**Published:** 2020-11-30

**Authors:** Lina Ke, Shusheng Yin, Shuting Wang, Quanming Wang

**Affiliations:** 1 School of Geography, Liaoning Normal University, Dalian, China; 2 National Marine Environmental Monitoring Center, Dalian, China; Institute for Advanced Sustainability Studies, GERMANY

## Abstract

Oceans and their resources are experiencing immense pressure because of human exploitation. The intensive use of sea areas has become an important method in solving the contradiction between ocean supply and demand, thereby ensuring sustainable marine economy development, tapping potential sea-area utilization, reasonably allocating sea-area utilization structures, and increasing marine economic benefits. This paper explores the definition and connotation of intensive sea-area use and constructs an evaluation index system based on marine input intensity, marine utilization structure, marine economic benefit, and marine ecological environment. Multi-objective variable fuzzy set theory and fuzzy decision analysis methods were used to evaluate the intensive sea-area utilization in the Liaoning Coastal Economic Zone of China during 2004–2016. The spatial differentiation characteristics of intensive sea-area use were analysed using cluster analysis. The research result showed that: (1) Intensive utilization level of the Liaoning coastal economic zone has gradually increased, while it is still in a moderately weak level; (2) Sea area intensive utilization varied in degrees and fluctuates in the six cities under the jurisdiction of the Liaoning coastal economic zone; and (3) Marine input intensity, marine utilization structure, marine economic benefit, and marine sustainability indexes have increased in the cities, thereby exhibiting improvements in the Liaoning coastal economic zone.

## 1 Introduction

China's marine space development is at a critical stage of transformation, and the material flow and energy exchange in the coastal zone are more frequent. The Man-sea relationship as an important part of man-land relationship [1, 2], Facing the problems of intensified marine economic activities and insufficient development space in coastal areas, it is also facing the pressure of marine environmental protection and marine space management. Therefore, in the new period of China's marine economic transformation and development, the rational development of marine space has become an important perspective and approach to promote the high-quality development of China's marine economy.

Under the current situation, high-quality development pressure in China's coastal areas highlights. With the increasing restriction of resources and environment, it is difficult to continue the traditional inefficient marine space development activities. In order to solve the problems caused by previous development activities, such as disorderly expansion of reclamation scale, waste of marine resources, decline of productivity of coastal biological resources, deterioration of water quality, siltation of waterway and biological invasion and many other problems [3–8]. In the "13th Five-Year Plan", the Chinese government clearly stated that "adhere to land and sea coordination, strengthen the marine economy, scientifically develop marine resources, and protect the marine ecological environment", and clearly defined the overall goal of "build China into a maritime power ", and the activities and forms of marine space development are being reconstructed. In this context, it is an important task to optimize the allocation of sea area utilization structure, improve the output of unit area, save and intensively use sea resources, which is an important topic to ensure the coordinated development of human and sea, promote the high-quality development of marine economy and solve the difficulties of space expansion and utilization in coastal areas.

In recent years, many domestic and foreign scholars have explored the connotation [9–12], research focus [13], utilization type [14–19], research methods [20–27], and the mechanism of the driving force [1, 30, 28–33] of intensive land use at different scales. These studies have provided a powerful basis and reference for the theory and method associated with investigating the intensive use of sea areas. The concept of “intensive sea use” first proposed in 2011 [34] and since then it has gradually gained application through relevant practical researches on the development and utilization of sea areas since then. Furthermore, research investigations have encompassed the impact of intensive sea use on marine ecology, marine resources, and coastal city economy [33–39]. Some researchers have evaluated the management and sustainability of future development of coastal habitats in Europe based on the disappearance of oyster reefs at the European coast/gulf [40, 41]. Lee et al. explored the effects of land reclamation on tides, water flow balance, deposition rate, and erosion rate at coastal zones in the Dongxi Island of Korea as the research area [42]. Luo et al. analysed the impacts of intensive sea projects on the marine ecological environment using the variation of the sea ecological environment comprehensive index. They developed a “habitat quality” and “ecological response” evaluation index system based on two aspects: non-biological and biological factors of marine ecosystems [38].

In general, as only a few studies on the intensive use of sea areas are available, analyses on the overall efficiency and potential of sea-area development and utilization are still lacking. These include studies on the assessment of the intensive use of sea area based on connotation, evaluation index systems, and evaluation methods. Therefore, based on the regional relationships between humans and the sea, this paper discusses the concepts and connotations of intensive sea use to construct an evaluation index system and to establish methods and technical routes to evaluate the intensive use of sea areas.

The highlight of this article is that this study will fill the gap in evaluating intensive sea use, which is a current resource use practice in China. Six prefecture-level cities in the coastal economic zone of Liaoning were used as the research area with the study period of 2004–2016. The research findings will provide a reference and basis for optimizing the spatial structure of sea-area utilization and regulating the scale of sea space development.

### 1.1 Concept and connotation of intensive use of sea area in a man-sea regional system

**Fig 1:**
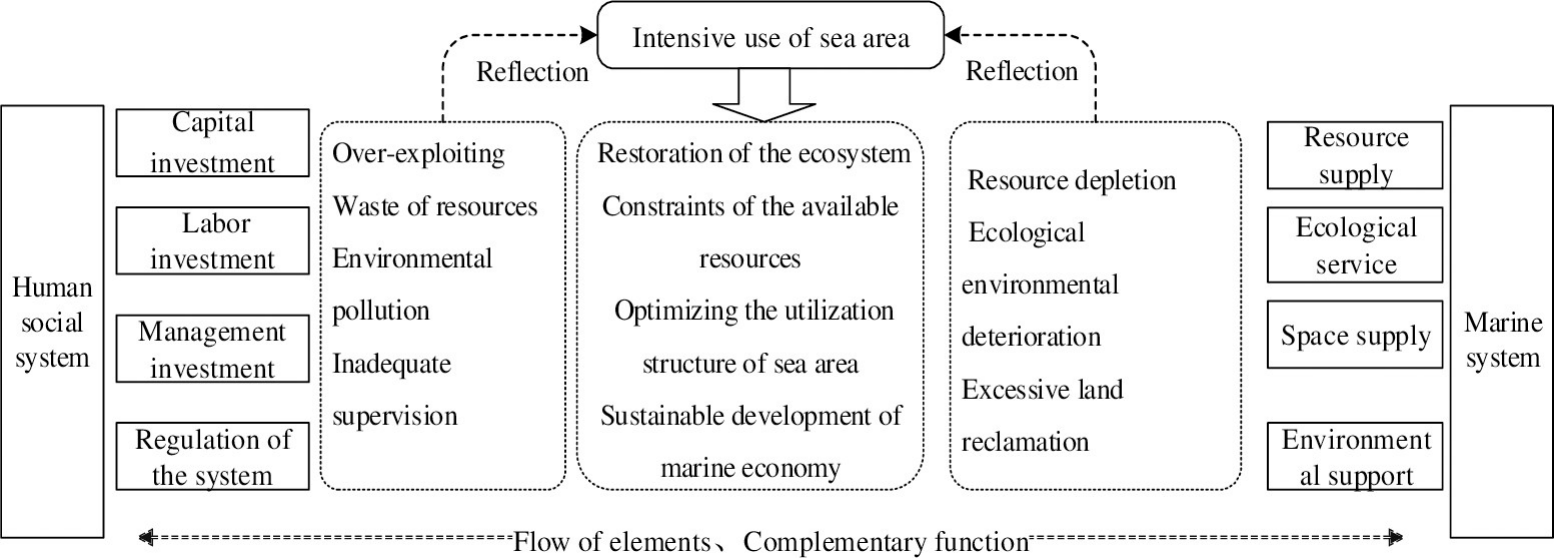
Analysis framework of intensive sea-area use based on the regional perspective of man-sea relationship.

The human-sea regional system is a geographical system composed of humans, the oceans, and land in one geographical area. This essentially reflects the mutual interaction between human society, sea, and land, i.e, humans on land developing and exploiting marine resources for their benefit by investing in various production factors such as labour, capital, and technology. This reflects the human cognition and control on the exploitation and utilization of marine resources and their participation, influence, and role in the development and evolution of the man-sea regional system. Humans lead the flow of system elements in a human-sea regional system and dominate human-sea interaction. However, the sea and land provide the basic elements (e.g, resources, space, and environment) necessary for human survival and development. This relationship improves the element flow and function of the human-sea regional system. Moreover, it reflects the mutual influence and response between human society and the natural environment. From the perspective of the regional system of human-sea relationship (Fig 1), different elements in the system interact and with it, impose constraints on each other. This complex relationship helps to maintain the sustainable development of the system due to the exchange of materials, energy, and information between the sea and land [1].

From the perspective of systematology, a system is always transitioning between disorder and order and equilibrium and non-equilibrium due to complex internal and external connections. The “coordination” between the human-sea regional system and its components can promote the system to change from disorder to order and further evolve towards a more complex level. However, overexploitation of marine resources results in wastes and pollution of the marine environment. These issues will further cause frequent marine disasters and depletion of resources, thereby ultimately constraining the sustainable development of the human-sea regional system. Therefore, to achieve sustainable development, it is necessary to address the uniqueness and integrity of the human-sea geographical relationship through the sea-land relationship by scientifically regulating the development of coastal activities, to promoting more rational, intensive, and highly efficient utilization of coastal resources, and establishing a coordinated development between humans and the sea in the coastal zones. These strategies will help realize an ideal configuration for space utilization in coastal zones and thus, integrating development between humans and the sea to promote marine ecological progress.

This paper attempts to utilize the definition and connotation of intensive sea-area use based on the man-sea regional system [1, 43–46] and intensive utilization [15–18, 23, 26]. It aims to improve the resource allocation efficiency, utilization rate, social benefits by increasing the factor of investment, management of resource utilization, and utilization structure at coastal areas. With these measures, the coastal resources can be used intensively with high efficiency to facilitate the development of coastal industries and promote the integration of land-sea economic and its sustainable development.

The intensive use of the sea area should not be limited to the definition of the sea area factor investment and economic benefit output. Instead, it should also consider the sustainability of the utilization and the coastal ecological environment. Sustainable development of marine ecology and growth of marine economy can only be achieved by developing and utilizing coastal resources under the premise of ecological environment protection. The dynamic and relative concept of intensive sea use represents marine economic development at an appropriate scale in a specific period and within a specific region under current or foreseeable future conditions and provides for processes for improving the utilization rate of coastal resources with moderating supply to sea areas to enhance economic output and social benefits from coastal resources. Therefore, the intensive use of sea areas can be defined as the process of improving overall marine benefits under existing constraints of marine resources and environmental conditions by (1) optimizing the utilization structure of sea areas, (2) improving the space management of sea areas, (3) increasing the utilization rate of coastal resources with moderate increase in supply, and (4) maintaining the health of marine ecosystem. The intensive sea use reflects multiple connotations including a more rational utilization structure, an improved economic benefit, and the sustainable utilization of sea areas.

## 2 Research method

### 2.1 Overview of research area and data sources

According to the “Guidance [47] on the overall planning of the comprehensive protection and utilization of provincial-level coastal zones”, a coastal zone is defined as an area that “covers the territorial administrative jurisdiction associated with the coastal county-level administrative regions and the external boundaries of the territorial sea within the jurisdiction of inland provinces (autonomous regions and municipalities). which “also consider the integrity and completeness of the adjacent ecosystems as well as the dependence of terrestrial economies on the oceans.” Therefore, the study explored the territorial administrative jurisdiction towards the land side along the coastal economic belt in Liaoning as the research area. This region is bound by the outer edge of the marine functional zones towards the seaside in the Liaoning Province. The coastal economic belt of Liaoning is in the north-eastern part of China and it is adjacent to the Bohai Sea and Yellow Sea. It encompasses six coastal cities (Dalian, Dandong, Jinzhou, Yingkou, Panjin, and Huludao) with a total sea area of 41,300 km^2^ and a total coastal line of 2,920 km including a 2,292.4-km coastline on the mainland side and a 627.6-km on the island coastline. The geographical location of the study area can be referred to https://www.tianditu.gov.cn/. During 2016, the Liaoning coastal economic belt generated a gross marine product of 1.14103 trillion RMB, which accounts for 50.86% of the gross provincial product. This contribution has also reached its maximum of 52.06% [48].

The data used in this study were obtained from multiple sources including statistical yearbooks, yearbooks, marine environmental status bulletins, survey data of sea area usage, and remote sensing image data of the Liaoning Province and its coastal cities. The area data of the sea, coastline length, total sea area, length of developed and utilized coastlines, and total amount collected from the sea-area utilization were obtained from the survey on the current utilization condition of the sea area and remote sensing image data. Environmental data, such as the comprehensive index of seawater quality and marine functional zoning conformity, were calculated from relevant data listed in the Liaoning Province Marine Environment Status Bulletin (2004–2016) [49]. The investment in fixed assets, total population, GDP, and other data were obtained from the Liaoning Statistical Yearbook (2004–2016) [50] and statistical yearbooks of various cities (2004–2016) [51–56] with changes of price index adjusted based on a constant price. The area of the marine protection zone spanning across multiple prefecture-level cities is divided according to the boundary of the administrative sea area. The data sources of this paper are shown in Table 1.

**Table 1:** Introduction of data and data sources.

Data	Data sources	Data links
Total collection Amount of sea area use fund	Bulletin on management of sea area use	http://gc.mnr.gov.cn/
	Remote sensing image	http://www.gscloud.cn/
The area data of the sea	Bulletin on management of sea area use	http://gc.mnr.gov.cn/
Length of coastlines	Bulletin on management of sea area use	http://gc.mnr.gov.cn/
Length of developed and utilized coastlines	Remote sensing image	http://www.gscloud.cn/
Marine functional zoning data	Marine functional zoning of Liaoning Province	http://zrzy.ln.gov.cn/
Socioeconomic data	Statistical bulletin of China's marine economy	http://gi.mnr.gov.cn/
	Liaoning statistical yearbook	http://www.ln.stats.gov.cn/
Remote sensing image	Landsat-8 OLI	http://www.gscloud.cn/
Marine functional zoning conformity	Bulletin of china marine ecological environment status	http://sthj.ln.gov.cn/
The comprehensive index of seawater quality	Bulletin of china marine ecological environment status	http://sthj.ln.gov.cn/
	Liaoning Province Marine Environment Status Bulletin	http://www.ln.gov.cn/

*Note*. all data used in this study were from 2004 to 2016

### 2.2 Evaluation of the intensive use of sea area

#### 2.2.1 Construction of an evaluation index system

Investment conditions, utilization structure, economic benefits, and sustainability are the main factors that determine the level of intensive use of a sea area. The definition and connotation of the intensive use of sea area was used as the basis and by applying the evaluation index system of intensive land use, combining the actual sea-area utilization condition in the Liaoning coastal economic belt, and utilizing the sea area extensively, the following aspects were measured: (1) investment intensity; (2) utilization structure, (3) marine economic benefit, and (4) sustainability of sea-area utilization.

1. The investment conditions of the various production factors provide the basis for sea-area utilization. The intensity of investment primarily measures the degree of capital, labour, resources, and other production factors in the sea area.
2. The sea-area utilization structure index accounts for the degree of balance and diversity index for using a specific sea area. It is used primarily to examine the utilization structure of different types of sea areas and reflects the structural allocation of resources, distribution of marine industries, and different types of utilization. A better understanding of the sea implies that it is exploited and utilized by humans to greater extents including better development of different sea areas [29, 31, 57]. The diversity of sea-area utilization provides a comprehensive measurement of the richness and complexity level. Hence, improving diversity in the utilized sea areas will help improve the level of intensive sea use.
3. The marine economic benefit index represents the transformation of marine resources into economic benefits. A higher economic benefit index indicates better production from marine resources and a higher level of intensive sea use. Marine economic benefit in this paper specifically refers to the gross marine production [57] including charges for using sea area as this is a paid utilization of marine resources.
4. Sustainable utilization of sea areas and their development inevitably affect the marine ecosystem adversely. Therefore, sustainability must be considered. The comprehensive index of water quality is a direct measurement of marine water quality. A smaller index value indicates a better marine ecological environment. Conformity to marine functional zoning is evaluated with the following criteria: compliance to target function, suitability of the approaches in utilizing the sea area, and satisfying environmental protection requirements [58, 59]. Based on the level associated with each criterion, the following five conformity levels are set: consistent, well compatible, compatible, conditionally compatible, and non-compliant. By following the principles of accessibility, science, dynamics, and pertinence, the evaluation index system of the intensive use of sea area is constructed, as shown in Table 2.

**Table 2:** Evaluation index system for intensive use of sea areas in the Liaoning coastal economic belt.

Criterion level	Indicator level	Weight	Definition and calculation of the index
Marine Input intensity	Fixed assets investment per unit sea area (RMB/km^2^)	0.259	Marine capital investment per unit area; Eq (1)
	Sea-area utilization rate (%)	0.264	Degree and rights conformance of sea-area development and utilization
	Shoreline development intensity	0.477	Extent of coastline development and utilization; artificial shoreline length/shoreline length
Sea-area Utilization structure	Structural balance of sea area	0.471	Structural level of sea-area development and utilization of structural level; Eq (2)
	Sea-area use type diversity index	0.529	Diversity of sea-area use types; Eq (3)
Marine economic benefits	Output value per unit sea area (RMB/km^2^)	0.312	Sea area output; Gross marine economic output/total sea area
	Marine economic contribution rate index (%)	0.379	Contribution of marine industry to the economy; GOP/GDP
	Collection of sea-area use fee (RMB /km^2^)	0.309	Paid use of sea area; Total collected and paid amount for sea-area use/ total used sea area
Sustainability of sea use	Comprehensive index of marine water quality	0.518	Water quality of sea area; water quality index of functional sea area was replaced by water quality index
	Marine function zoning compliance (%)	0.482	It can be divided into five grades: consistent, good compatibility, compatibility, conditional compatibility, and non-conformity

Notes: (1) Based on the research method by Wang and Han [60], fixed assets investment per unit sea area = total marine fixed assets investment / sea area. Total marine fixed assets investment = total investment in fixed assets in coastal areas × (gross marine product / gross regional product).

(2) The degree of sea-area utilization balance is calculated by the following equations:

$$\begin{array}{l} G=-\sum \left(P_{i}\ln P_{i}\right)\\ P_{i}=\frac{S_{i}}{\sum S_{i}}(i=1,2\ldots n)\\ J=\frac{G}{G_{\max}}=\frac{G}{\ln n}=\frac{\sum \left(P_{i}\ln P_{i}\right)}{\ln n} \end{array}$$

where *G* is the information entropy; *S* is the total utilized sea area; *S*_*i*_ is the sea area utilized under a different category; *P*_*i*_ is the fraction of sea area utilized under each category; *n* is the total number of categories for sea-area utilization; *J* is the degree of sea-area utilization balance. The information entropy is maximized when different categories of sea use share the same area, i.e, *G*_*max*_ *= ln n*.

(3) The utilization diversity of sea area is calculated by the equation: $Gm=1-\left(\sum_{i=1}^{n}x^{2}i/{\left(\sum_{i=1}^{n}x_{i}\right)}^{2}\right)$, where *i* is the utilization category of the sea area, *x*_*i*_ is the sea-area used by the *i*th utilization category, *n* is the total number of categories for sea-area utilization, and *G*_*m*_ is the utilization diversity of sea area. A larger *G*_*m*_ represents more diverse methods of sea-area utilization.

(4) Shoreline development intensity: artificial shoreline length/shoreline length.

(5) The conformity of marine function zone planning reflects the connection between marine function zoning and sea area use. According to the degree, it is divided into five grades: consistent, well compatible, compatible, conditionally compatible, and nonconforming.

#### 2.2.2 Assigning levels to the evaluation index

Currently, there is no universal classification standard for evaluating the intensive use of sea areas. By applying relevant literature [61] to the Liaoning coastal economic zone, this paper proposes physical factors that affect the evaluation results of the intensive use of sea areas such as resources and environment, which is comparable to the effects of pollution factors to environmental quality. Using the calculation of the Weber–Fechner law (W–F) shown in Eqs (1) and (2), the range associated with the standards of each evaluation index level can be determined. This method can also obtain the classification criteria of the indices for evaluating the intensive use of sea areas.

The *i*th index can be classified into *n* levels. The value associated with level *k =* 1 is *m*_*i0*_ and the value associated with level *k =* n is *m*_*id*_. With this, the objective ratio between any two levels *k* and *l* of the *i*th index can be obtained as:

$$\frac{m_{ik}}{m_{il}}=a^{k-1}\left(k,l=1,2,\cdots ,n\right)$$ (1)

where, *a*_*i*_ is the objective impact ratio between two adjacent levels of the *i*th index, calculated as:

$$ai={\left(m_{id}/m_{i0}\right)}^{1/n}$$ (2)

#### 2.2.3 Evaluation model and method

Variable fuzzy evaluation [62, 63] can combine certainty and uncertainty into a single system to provide a comprehensive evaluation; thus, providing a new idea and method for the multi-index and multi-level comprehensive evaluation problems. This study introduces this method to evaluate the intensive use of sea areas. Specifically, a variable fuzzy evaluation model based on the multi-objective variable fuzzy set theory is constructed for the assessment. The evaluation objects are identified according to *m* indices and *n* levels. The final result is represented by a characteristic level value *h* (*h* = 1, 2, 3,.., n), which represents different levels of the intensive use of sea areas (extensive use, low intensive use, moderate intensive use, high intensive use, and delicate intensive use). With this, the object sample matrix and index standard matrix associated with the evaluation are given by:

$$X=(x_{ij})\;i=1,2,3,\ldots ,m;\;j=1,2,3\ldots ,n$$ (3)

$$Y=(y_{ih})\;i=1,2,3,\ldots ,m;\;h=1,2,3\ldots ,n$$ (4)

To unify the dimension and to standardize the index sample matrix and the index standard characteristic value matrix, the following calculation is further performed:

$$rij=\left\{\begin{array}{l} 0,xij<yic(Negative-efficacy-indicator),\;xij>yic,\left(positive-efficacy-indicator\right)\\ \frac{xij-yic}{yi1-yic},yi1>xij>yic,yi1<xij<yic\\ 1,xij<yi1\left(Negative-efficacy-indicator\right),\;xij>yi1,\left(positive-efficacy-indicator\right) \end{array}\right\}$$ (5)

$$Sih=\left\{\begin{array}{l} 0,yih<yic\left(Negative-efficacy-indicator\right),yih>yic\left(positive-efficacy-indicator\right)\\ \frac{yih-yic}{yi1-yic},yi1>yih>yic,or,yi1<yih<yic\\ 1,yih\geq yi1\left(positive-efficacy-indicator\right),\;yih<yi1,(Negative-efficacy-indicator) \end{array}\right\}$$ (6)

where, *r*_*i*_ is the characteristic value membership of sample *j* and index *i* in the evaluation of the intensive sea-area use; *s*_*ih*_ is the standard normalized index of the *i*th evaluation index with respect to the characteristic level *h* of the intensive sea-area use.

Next, Eq (7) is then used.

$$u_{hj}=\left\{\begin{array}{l} 0,1\leq h<a_{j},c\geq h>b_{j}\\ {\left\{\sum_{k=a_{j}}^{b_{j}}{\left[\frac{\sum_{i=1}^{m}{\left[w_{i}\left|r_{ij}-s_{ih}\right|\right]}^{p}}{\sum_{i=1}^{m}{\left[w_{i}\left|r_{{}_{ij}}-s_{{}_{ik}}\right|\right]}^{p}}\right]}^{\frac{a}{p}}\right\}}^{-1},a_{j}<h<b_{j} \end{array}\right\}$$ (7)

Where, *a*_*j*_ is the lower limit and *b*_*j*_ is the upper limit of the evaluation level *j* for intensive sea-area use. Values for *α* and *p* can be selected from four combinations: *α =* 1 or 2 and *p =* 1 or 2. Each combination is used under different conditions representing the linear and nonlinear relationship between the level index and level characteristic value.

Therefore, the relative membership matrix of the evaluation object of intensive sea-area use with respect to the evaluation level standard can be constructed as:

$$U=(u_{hj})\;h=1,2,3\ldots ,n;\;j=1,2,3\ldots ,m$$ (8)

The level characteristic value model and its evaluation sample set can be obtained based on previous literatures [62, 63]. These values are then used as the intensive sea-area utilization index. The level characteristic value of the evaluation sample set is given by

$$H=(1,2,3\ldots ,n)u_{hj}$$ (9)

### 2.3 Determining the evaluation method based on the weights

This study used the fuzzy decision analysis theory to determine the weight of each index [57]. In the fuzzy decision analysis theory, the matrix rows and numbers are arranged from large to small according to the sorting consistency. This operation provides the order of importance of the target set. The target set is then first sorted by binary comparison in the order of importance and then normalized. Finally, the target weight vector for each index can be obtained as:

$$\begin{array}{l} w=\left\{\beta_{1}/\sum_{i=1}^{m}\beta_{i},\beta_{2}/\sum_{i=1}^{m}\beta_{i},\ldots \ldots \beta_{m}/\sum_{i=1}^{m}\beta_{i}\right\}\\ \;=\left\{\beta_{1}/\sum_{i=1}^{m}\sum_{j=1}^{m}\beta_{ij},\beta_{2}/\sum_{i=1}^{m}\sum_{j=1}^{m}\beta_{ij},\ldots \beta \sum_{i=1}^{m}\sum_{j=1}^{m}\beta_{ij}\right\},i\neq j \end{array}$$ (10)

where, *β*_*ij*_ is the fuzzy importance scale of target *i* with respect to *j* (mood operator).

## 3 Results and analysis

### 3.1 Analysis on the variations of comprehensive index of intensive sea-area use

This study evaluated the intensive utilization level at the Liaoning coastal economic belt based on the multi-objective variable fuzzy method. The evaluation results of different cities are shown in Table 3. The levels were obtained through K-means clustering analysis using the SPSS software. The intensive sea-area utilization condition at the Liaoning coastal economic zone is divided into five levels. Table 4 shows the type and range of each level with the number of intensive use levels in Table 4.

**Table 3:** Intensive utilization degree and grade of sea area in Liaoning coastal economic zone.

Year	Dalian		Dandong		Yingkou		Panjin	
	Intensive index	Level	Intensive index	Level	Intensive index	Level	Intensive index	Level
2004	2.40	Ⅰ	2.67	Ⅰ	3.15	Ⅱ	3.25	Ⅲ
2005	2.54	Ⅰ	2.65	Ⅰ	3.34	Ⅲ	3.54	Ⅲ
2006	2.46	Ⅰ	2.95	Ⅱ	3.58	Ⅳ	3.52	Ⅲ
2007	2.90	Ⅱ	3.00	Ⅱ	3.61	Ⅳ	3.51	Ⅲ
2008	2.98	Ⅱ	2.93	Ⅱ	3.68	Ⅳ	3.72	Ⅳ
2009	3.05	Ⅱ	3.18	Ⅱ	3.89	Ⅴ	3.83	Ⅳ
2010	3.03	Ⅱ	3.27	Ⅲ	3.99	Ⅴ	3.75	Ⅳ
2011	3.15	Ⅱ	3.27	Ⅲ	4.02	Ⅴ	3.76	Ⅳ
2012	3.31	Ⅲ	3.44	Ⅲ	3.87	Ⅴ	3.83	Ⅳ
2013	3.34	Ⅲ	3.49	Ⅲ	4.04	Ⅴ	3.97	Ⅴ
2014	3.56	Ⅲ	3.39	Ⅲ	4.10	Ⅴ	3.88	Ⅴ
2015	3.67	Ⅳ	3.46	Ⅲ	4.18	Ⅴ	3.94	Ⅴ
2016	3.77	Ⅳ	3.52	Ⅲ	4.27	Ⅴ	3.99	Ⅴ
Year	Jinzhou		Huludao		Liaoning coastal economic belt			
	Intensive index	Level	Intensive index	Level	Intensive index	Level		
2004	2.86	Ⅱ	2.51	Ⅰ	2.81	Ⅱ		
2005	3.03	Ⅱ	2.60	Ⅰ	2.95	Ⅱ		
2006	3.31	Ⅲ	2.61	Ⅰ	3.07	Ⅱ		
2007	3.42	Ⅲ	2.76	Ⅰ	3.20	Ⅲ		
2008	3.65	Ⅳ	2.91	Ⅱ	3.31	Ⅲ		
2009	3.65	Ⅳ	3.13	Ⅱ	3.45	Ⅲ		
2010	3.83	Ⅳ	3.35	Ⅲ	3.54	Ⅲ		
2011	3.93	Ⅴ	3.51	Ⅲ	3.61	Ⅳ		
2012	3.89	Ⅴ	3.45	Ⅲ	3.63	Ⅳ		
2013	3.74	Ⅳ	3.50	Ⅲ	3.68	Ⅳ		
2014	3.96	Ⅴ	3.26	Ⅲ	3.70	Ⅳ		
2015	4.06	Ⅴ	3.33	Ⅲ	3.78	Ⅳ		
2016	4.14	Ⅴ	3.40	Ⅲ	3.86	Ⅴ		

**Table 4:** Grading types of intensive utilization of sea area.

Range	Level of intensive utilization	Type	Amount
0–2.79	Extensive	Ⅰ	9
2.79–3.19	Low intensive	Ⅱ	17
3.19–3.56	Moderate intensive	Ⅲ	30
3.56–3.85	Highly intensive	Ⅳ	18
3.85–5	Fine use	Ⅴ	17

**Fig 2:**
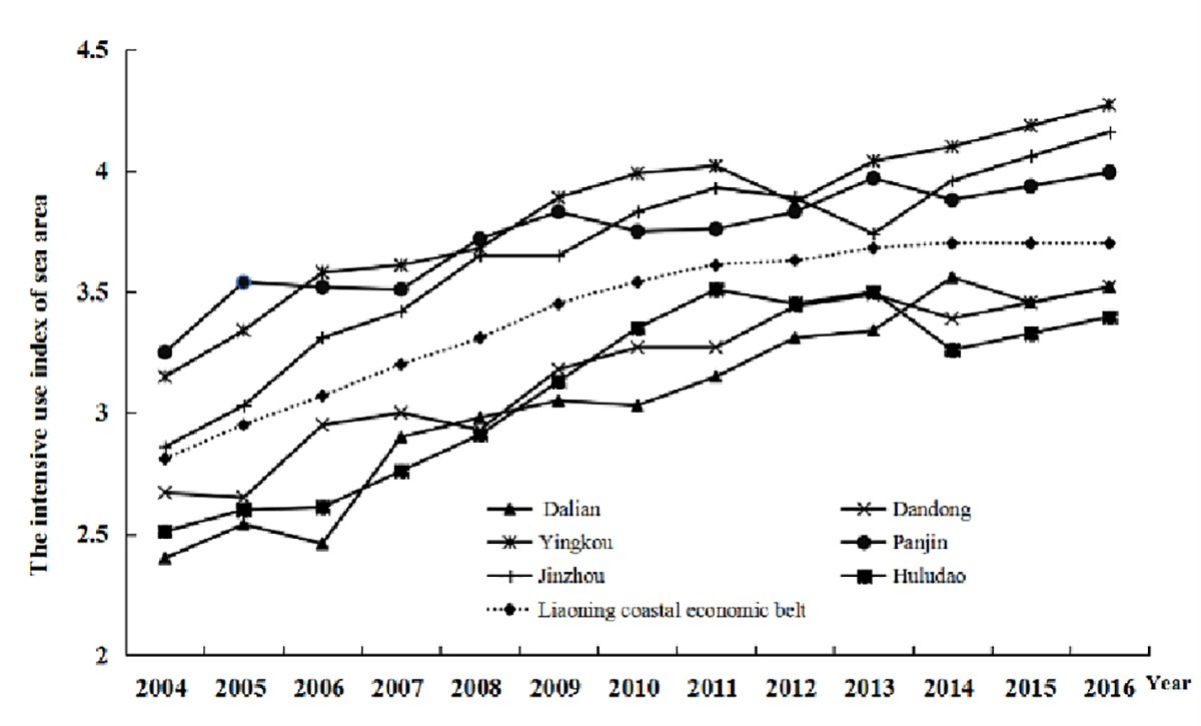
Trends of the intensive use of sea areas.

As shown in Table 3, the level of intensive sea-area use at the Liaoning coastal economic belt continuously increased during 2004–2016. However, this level is still in the moderately intensive level. Medium and poor use of intensive sea areas accounted for 61% of the total evaluation samples. This shows that the intensive utilization level still needs improvement. Fig 2 shows a plot of the intensive use trend map at the Liaoning coastal economic belt during 2004–2016 showing the difference in the intensive use level in the different cities. The intensive use level continued to rise from 2004 to 2016; however, the rate of development varied between the six cities. The SPSS cluster analysis classified the annual average growth rate of the intensive sea-area utilization index by three types: fast-growing, steady growing, and slow growing (as shown in Table 5).

**Table 5:** Increasing types of intensive utilization of sea area.

Growing type	Cities	Annual growth rate (%)
Fast growing	Dalian	3.81
	Jinzhou	3.01
Steady growing	Huludao	2.44
	Yingkou	2.42
Slow growing	Dandong	2.18
	Panjin	1.16

Dalian is an important city in the Liaoning coastal economic belt with its well-developed economy and rich marine resources. Its sea area has been diversely utilized including for fishing, industrial, transportation, and tourism/entertainment use. Fishery is the major type of sea-area utilization in Dalian and it is developed primarily based on the fishing resources around the Changshan islands. The industrial use of the sea areas can be found in almost all the counties in Dalian except for the Changhai county. During the study period (2004–2016), the comprehensive index of intensive sea-area use in Dalian increased from 2.40 to 3.77 with an average annual growth rate of 3.81%. This represents the fastest growth among all the cities of the Liaoning coastal economic belt. By analysing the different levels of indices in Table 6, both the sea-area utilization structure and marine economic benefit indexes increased steadily from 2004 to 2016 in Dalian, thereby leading to the increase of its comprehensive index of intensive sea-area use and a rapid increase in its intensive use level of sea area. The large sea area and long coastal line of Dalian both resulted in a relatively low level of investment intensity in fixed assets, shoreline development intensity, and utilization rate per unit sea area. As a result, the intensive utilization level of sea areas in Dalian is still at a moderate level even with its rapid rise with plenty of room for future improvements.

**Table 6:** Evaluating the value of the standard layer of intensive sea-area use.

Year	B_1_ Marine input intensity						
	Dalian	Dandong	Yingkou	Panjin	Jinzhou	Huludao	Liaoning coastal economic belt
2004	1.18	2.6	4.13	3.99	3.32	1.93	2.86
2005	1.61	2.68	4.31	4.04	3.46	2.06	3.03
2006	1.71	2.74	4.43	4.09	3.53	2.18	3.11
2007	1.82	2.86	4.65	4.22	3.68	2.35	3.26
2008	1.91	2.97	4.72	4.34	3.79	2.42	3.36
2009	2.00	3.09	4.75	4.50	3.89	2.63	3.48
2010	2.08	3.24	4.81	4.71	4.12	2.87	3.64
2011	2.17	3.26	4.85	4.61	4.21	2.98	3.68
2012	2.26	3.33	4.89	4.69	4.27	3.13	3.76
2013	2.29	3.42	4.92	4.80	4.28	3.18	3.97
2014	2.32	3.34	4.95	4.79	4.32	3.23	3.83
2015	2.42	3.41	4.98	4.86	4.41	3.35	3.92
2016	2.53	3.47	5.05	4.94	4.50	3.47	4.01
Year	B_2_ Sea-area utilization structure						
	Dalian	Dandong	Yingkou	Panjin	Jinzhou	Huludao	Liaoning coastal economic belt
2004	2.63	1.93	2.85	2.22	2.13	1.75	2.25
2005	2.42	1.80	2.82	2.22	2.16	1.76	2.20
2006	2.13	2.40	3.15	2.22	2.20	1.7	2.30
2007	2.85	2.40	2.54	2.22	2.15	1.62	2.30
2008	2.95	2.19	2.68	2.22	2.13	1.8	2.33
2009	2.88	2.40	2.92	2.22	2.11	1.83	2.39
2010	2.76	2.21	2.90	2.20	2.10	1.79	2.33
2011	2.60	2.29	2.92	2.22	2.10	1.79	2.32
2012	2.91	2.30	2.71	2.22	1.95	1.73	2.30
2013	2.57	2.27	2.95	2.22	2.01	1.58	2.27
2014	2.99	2.24	2.83	2.22	2.33	1.43	2.34
2015	3.02	2.27	2.86	2.22	2.35	1.44	2.35
2016	3.06	2.30	2.88	2.22	2.37	1.48	2.36
Year	B_3_ Marine economic benefits						
	Dalian	Dandong	Yingkou	Panjin	Jinzhou	Huludao	Liaoning coastal economic belt
2004	1.93	1.92	1.25	1.80	1.27	1.26	1.57
2005	1.91	1.21	2.06	2.08	1.32	2.01	1.76
2006	2.23	2.65	2.19	2.36	1.35	2.32	2.18
2007	2.23	2.35	2.46	2.46	1.39	2.44	2.22
2008	2.24	2.70	2.51	2.60	2.49	2.58	2.52
2009	2.28	2.41	2.72	2.73	2.53	2.62	2.55
2010	2.38	2.64	2.72	2.80	2.48	2.74	2.63
2011	2.41	1.67	2.79	2.81	2.69	2.78	2.52
2012	2.37	2.76	2.86	2.54	2.68	2.67	2.65
2013	2.53	2.91	2.91	2.68	1.76	2.93	2.62
2014	2.60	1.87	2.95	2.82	2.87	1.95	2.51
2015	2.66	1.87	3.10	2.91	3.02	2.01	2.60
2016	2.72	1.86	3.26	3.01	3.16	2.08	2.68
Year	B_4_ Sustainability of sea use						
	Dalian	Dandong	Yingkou	Panjin	Jinzhou	Huludao	Liaoning coastal economic belt
2004	2.57	2.56	1.43	1.60	1.04	3.14	2.06
2005	2.57	2.56	1.85	1.60	1.67	3.14	2.23
2006	2.57	2.56	1.85	1.60	2.29	3.14	2.34
2007	2.57	2.56	1.85	1.60	2.29	3.14	2.34
2008	2.57	2.56	1.52	1.60	2.71	2.72	2.28
2009	2.57	2.56	1.52	1.60	2.29	3.14	2.28
2010	2.57	2.56	2.08	1.60	2.71	3.14	2.44
2011	2.57	2.56	2.08	1.60	2.71	3.14	2.44
2012	2.57	2.56	1.52	1.60	2.71	2.72	2.28
2013	2.57	2.56	1.52	1.63	2.26	2.88	2.23
2014	2.61	2.56	2.35	1.60	2.71	3.03	2.48
2015	2.61	2.56	2.43	1.60	2.86	3.02	2.52
2016	2.62	2.56	2.52	1.60	3.01	3.01	2.56

The sea area in Jinzhou accounts for 3% of the total utilized sea area in Liaoning. Jinzhou has been focusing on fishing, transportation, and tourism/entertainment usage of its sea area. Aquaculture in an open system is the leading sea use in Jinzhou. Regional land reclamation, which is primarily used for the construction of the Jinzhou Port and Jinzhou World Expo Park, also contributed to a significant portion of sea-area use in Jinzhou. During the study period (2004–2016), the comprehensive index of intensive sea-area use in Jinzhou increased from 2.86 to 4.16 with an average annual growth rate of 3.01%. By analysing the different levels of indices in Table 6, the investment intensity, and the sustainability of sea-area utilization increased substantially from 2004 to 2016 in Jinzhou. For example, the investment in fixed asset per unit sea area in Jinzhou is only 5.8524 million RMB/km^2^ in 2004 and it has risen to 78.8567 million RMB/km^2^ in 2016. This represents a significant growth in the sea area investment intensity. In addition, Jinzhou has dedicated considerable efforts in rectifying and renovating its coastal zones, which has resulted in the continuous improvement of the marine environmental quality and the continuous increase of its Level 1 and Level 2 seawater. Jinzhou reached 100% compliance with marine functional zones by 2016. In general, Jinzhou experienced a continuous increase in its intensive sea-area use as reflected by the rising utilization structure index of sea area.

The sea area in Yingkou has also been utilized in different ways including fishing, industrial, transportation, tourism/entertainment, and special use. Particularly, the transportation use of sea areas in Yingkou, primarily strengthened by the construction of the Bayuquan and Xianrendao Ports, accounted for a high fraction of the total transportation use in Liaoning. A strong investment intensity and high utilization structure index of sea area have contributed to the increasing marine economic benefit index in Yingkou and thereby, it maintains a high level of the comprehensive index of intensive sea-area use among different cities of the Liaoning coastal economic zone. During the study period (2004–2016), the comprehensive index of intensive sea-area use in Yingkou increased from 3.15 to 4.27 with an annual growth rate of 2.42%. However, due to the ecological and environmental issues caused by intense marine development, the sustainability of sea-area utilization in Yingkou has remained at a relatively low level exhibiting a 33.3% compliance rate for the marine functional zones at nearshore sea area in Yingkou from 2011–2016. This suggests that sub-healthy and unhealthy conditions prevail in the local marine ecosystems of the city.

From 2004 to 2016, the comprehensive index of intensive sea-area use in Huludao has increased from 2.51 to 3.40 with an annual growth rate of 2.44%. However, the comprehensive index of intensive sea-area use in Huludao has remained relatively low mainly due to the low utilization structural index of the sea area in the city. The total usable sea area in Huludao is 2.1 × 10^9^m^2^. These areas have undergone limited utilization such as land reclamation for industrial and county construction, transportation from the port, and aquaculture. In addition, the resource utilization at the coastal zone in Huludao also suffers from poor utilization efficiency and idling issues. This improper resource utilization of sea area is the primary cause of the low utilization structure index and low marine economic benefit index in Huludao; thus, preventing the improvement of its intensive utilization level of sea area.

The increase in the comprehensive index of intensive sea-area uses in Dandong and Panjin is relatively slow. The intensive utilization level of sea area in Panjin is greater than that in Dandong. In 2016, Panjin reached the delicate use category of the intensive utilization level of the sea area. Contrarily, Dandong experienced a relatively poor intensive utilization level of sea area with a slow growth rate. Specifically, its comprehensive index of intensive sea-area uses only increased by 0.85 from 2004 to 2016. The economy of Dandong is relatively less developed among all the cities at the Liaoning coastal economic belt. This is primarily due to (1) the small utilization scale (6.3×10^8^ m^2^) of its sea area and (2) the limited utilization approach of its sea area dominated by aquaculture and fishery over a long period. For example, the sea-area used for aquaculture and fishery accounted for 92% of the total sea area usage in 2004 and it has dropped to 70% in 2016 as port transportation and industrial use contributed to 20% and 7% of the total sea-area use, respectively. Even with slight transformations of the marine economic structure in Dandong, its utilization structure index of sea area still remains relatively low, thereby hindering the rapid marine economic development in Dandong with a low comprehensive index of intensive sea-area use.

### 3.2 Analysis of the intensive sea-area use based on the indices for standard layers

**Fig 3:**
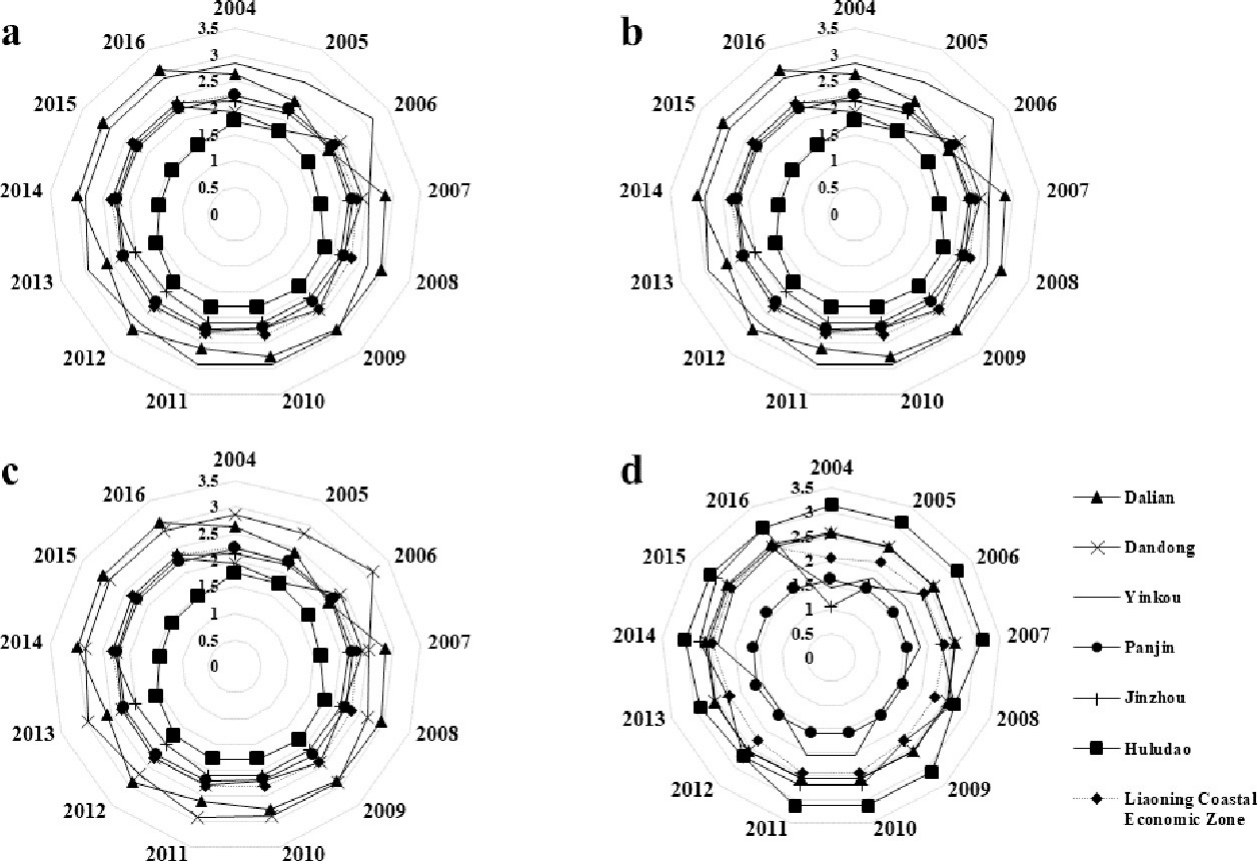
Standard layer index of intensive utilization of sea area: (a) Marine input intensity, (b) Sea-area utilization structure, (c) Marine economic benefits, and (d) Sustainability of sea use.

In this study, the evaluation index system for intensive sea-area use is divided into four standard layers. The evaluation values for each layer shown in Table 6 can be calculated using the aforementioned methods. Using these values, the variation trend of the indices for each standard layer in the evaluation of intensive sea-area use can be plotted (Fig 3).

The intensity index of investment in sea areas (Fig 3A) steadily increased from 2004 to 2016 in the Liaoning coastal economic belt and its cities. Different cities share a similar growth rate. In terms of rank (from high to low order) the cities are ordered as Yingkou, Panjin, Jinzhou, Dandong, Huludao, and Dalian. The rapid development of the society and economy in the Liaoning coastal economic belt has promoted faster marine development, thereby increasing the investment in fixed assets annually, increasing the utilized sea area, and intensifying development along the coastal line. These factors contribute to the continuous rise of the intensity index of investment.

The utilization structure index of sea area (Fig 3B) has only increased slightly by 0.11 along the Liaoning coastal economic belt. This is attributed to the increased sea area utilized for constructing coastal ports and industrial towns in Liaoning during 2004–2014. The sea-area utilization balance has slightly improved, but the utilization structure index of sea area has slowly increased. For future development of marine economy, adjustments of the marine industrial structure should be considered for proper utilization of the space and resources in the sea area. The utilization structure indices of the sea area for the six cities in the Liaoning coastal economic belt followed a similar variation trend. For Dalian, Yingkou, and Jinzhou, their high economic development level resulted in greater demand and diversity of sea use. Therefore, their utilization structure index of sea area remained relatively high with a continuous and rapid increase. Fluctuations were visible in the utilization structure index of sea area in Yingkou and Dalian, whereas that of Panjin remained relatively stable from 2004 to 2016. The utilization structure index of sea area in Huludao is relatively low with the dominant sea use being engineering construction. Due to the slow restructuring of the marine industry and limited utilization approaches of the sea area, the sea-area use in Huludao experienced a low degree of balance in terms of utilization structure and poor diversity in terms of utilization approaches. This shows the need for greater diversity in utilizing the sea areas in Huludao.

The marine economic benefit of the Liaoning coastal economic belt (Fig 3C) followed a fast-growing trend. In particular, the marine economic benefit index in Jinzhou experienced the fastest growth along with large fluctuations. By analysing each index, the fee for sea-area use collected by Jinzhou fluctuated significantly during the study period, thereby affecting its marine economic benefit index. This reflects the transformation and development of the marine economic structure of Jinzhou City during the evaluation period.

The sustainable index of sea-area utilization in the Liaoning coastal economic belt (Fig 3D) increased by 0.50 from 2004 to 2016. This demonstrates an improvement in the marine ecological environment in the Liaoning coastal economic belt. Among the six cities, Panjin and Yingkou exhibited a low sustainable index of sea-area utilization, which is mainly caused by their locations at the estuary of the Liao river, where the pollution from the land sources is most severe. Moreover, oil pollution caused by the exploitation of marine oil and gas resources is prominent in these cities. Finally, the sea areas of both the cities are semi-enclosed shallow bays, where the exchange capacity of the water body between the nearshore and sea is quite weak. Therefore, these features hinder the improvement of the sustainable index of sea-area use in Panjin and Yingkou.

## 4 Conclusions

This paper discussed the definition and connotation of intensive sea-area use. Based on the definition and connotation, a comprehensive evaluation index system was developed to assess the intensive sea-area use. A multi-objective fuzzy decision analysis method was used to analyse the intensive utilization condition and various index layers of the Liaoning coastal economic belt from 2004 to 2016. The primary conclusions drawn from this study are given as follows:

1. The intensive utilization level of sea area in the Liaoning coastal economic belt continually increased from 2004 to 2016. However, this intensive utilization level of sea area remains at a moderate developing level with plenty of room for future improvement.
2. The rate of development in the intensive utilization level of sea areas fluctuated and differed for the six cities in the Liaoning coastal economic belt. In terms of intensive utilization level of sea areas, Jinzhou and Dalian are fast-growing cities with an average annual growth rate of more than 3%, whereas Yingkou and Huludao are steadily growing. Yingkou has a significantly higher intensive utilization level of sea area than that of Huludao. Dandong and Panjin are slowly growing cities in terms of the intensive utilization level of sea areas with the lowest growth displayed by Dandong.
3. The intensity index of investment in the sea area increased steadily in the Liaoning coastal economic belt and its cities. Particularly, the sea area investment intensities in Yingkou and Panjin are relatively high. The utilization structure index of the sea area increased at a relatively slow rate. Among all the cities, Dalian and Yingkou possessed relatively high and highly fluctuating growth rates in terms of the utilization structure index of the sea area, while that in Huludao is relatively low. Furthermore, the marine economic benefit index demonstrated a fast-growing trend with a particularly high growth rate from 2004 to 2010. This index is found to increase the fastest in Jinzhou along with the largest magnitude of fluctuation. The sustainable index of sea-area utilization is found to change over different stages. Among all the cities, Panjin and Yingkou exhibited low sustainable index values of sea-area utilization.

## 5 Discussion

The intensive use of sea area is closely related to the transformation of national marine regional development strategy, corresponding to the transformation of marine economic development stage. This paper attempts to analyze the connotation of the intensive use of sea area from the perspective of intensive utilization, and construct the evaluation system of intensive utilization of sea area, the results show that the development level of marine economy and the quality of marine ecological environment are the important factors limiting the improvement of the level of intensive utilization of sea areas. The research results of this paper can provide a certain basis for the compilation and implementation of marine spatial planning and the promotion of high-quality development of marine economy. In addition, this paper identifies the temporal and spatial differentiation characteristics of the intensive use of sea areas in the Liaoning Coastal Economic Zone, which helps to make up for the lack of current research on the intensive use of marine space in China.

It needs to be pointed out that as an open and complex regional system, the intensive utilization of sea area is a systematic proposition involving nature and society, internal and external, process and results, mutation and adaptations. The influencing factors and mechanism involved are complex and changeable. However, this article is limited to the availability of data and the difficulty of quantifying maritime control policies. The evaluation indicators for intensive sea areas are not yet comprehensive and complete. How to scientifically select indicators to fully and accurately reflect the characteristics of intensive use of sea areas will be the focus of the next step. In addition, with the continuous strengthening of marine space management and control measures, various sea-use quotas are controlled by the government, and human intervention is continuously increasing. It is urgent to reveal the internal connection between sea area utilization and national control [64]. Relevant studies also show that there are obvious inefficiencies in China's marine economy, and the research area in this paper is just one of the typical regions [65], Therefore, the issues discussed in this paper need to be further expanded, expand the research perspective, deepen the understanding of the law of intensive use of sea areas in the process of marine development activities, so as to provide more targeted reference suggestions for marine space management and control in the context of high-quality development of marine economy.

This study explored and analysed the spatiotemporal variation characteristics of the intensive sea-area use in the Liaoning coastal economic zone from 2004 to 2016. These analyses can serve as a basis for formulating major marine-function zonation and in implementing policies related to sea area management. However, the proposed evaluation index system needs to be further improved due to the constraints of limited marine economic and social-ecological data. Currently, it is still difficult to analyse the spatiotemporal differences in the intensive sea-area use and the mechanism of their evolution in depth. Methods for improving intensive sea-area use were not discussed in this paper and they can be investigated in future studies.

In addition, it should be noted that the evaluation of intensive sea-area use should not follow exactly the same model used for evaluating intensive land-area use. Compared with the research studies on intensive land-area use, the use of sea areas possesses distinctive features such as an easily adjustable structure and an open marine environment [64]. Therefore, the evaluation index system should reflect these unique features associated with sea-area use. On the other hand, for most regions in China, there is still a huge potential for further development of the sea area [65], and substantial room exists for further optimization of sea use configuration. Future research is of great significance in promoting the coordinated development of coastal land resources.
